# Commentary on “Body mass index, waist circumference and mortality in subjects older than 80 years: a Mendelian randomization study” by Lv *et al*

**DOI:** 10.1093/eurheartj/ehae295

**Published:** 2024-06-28

**Authors:** Stephen Burgess, Ang Zhou

**Affiliations:** 1 MRC Biostatistics Unit, University of Cambridge, Cambridge, UK; 2 Cardiovascular Epidemiology Unit, Department of Public Health and Primary Care, University of Cambridge, Cambridge, UK; 3 Australian Centre for Precision Health, Unit of Clinical and Health Sciences, University of South Australia, Adelaide, Australia

In their paper, Lv *et al* examine the relationships of body mass index and waist circumference with mortality outcomes in a dataset of elderly Chinese individuals using conventional and genetic epidemiological approaches (1). They show that higher body mass index (BMI) and higher waist circumference are associated with lower risk of mortality in observational analyses. In genetic analyses, higher genetically-predicted BMI was associated with lower risk of mortality, but higher genetically-predicted waist circumference was associated with higher risk of mortality. These results differ from most previous investigations into the relationship between body size and mortality (2). However, on closer inspection, there are clear reasons that lead to these discrepancies. First, the population in this study is elderly, with all participants being over 80 years and the mean participant age at 90.6 years. Second, the distribution of BMI is low, with over 40% of participants having a BMI below 18.5 kg/m^2^. While obesity is generally associated with higher mortality risk, several observational analyses have reported the lowest level of disease risk around 25 kg/m^2^ (3), which is towards the top end of the BMI distribution in the Chinese dataset. Hence, while this analysis indicates that high BMI is good and other analyses have indicated that high BMI is bad, the overall observational picture is a U-shaped relationship for middle-aged healthy populations. This analysis provides important information to aid prediction of mortality in underweight elderly individuals, who will differ from middle-aged populations not only in the population distribution of body mass index, but also in the ratios of lean mass and muscle mass to fat mass.

While in some cases, genetic epidemiological analyses (such as the Mendelian randomization analyses presented by Lv *et al*) enable researchers to make causal conclusions, in this case we would be sceptical about a causal interpretation of the genetic findings. A causal claim relies on the genetic variants influencing the exposure in a specific way such that any causal pathway from the genetic variants to the outcome passes via the risk factor (4). When assessing the validity of a Mendelian randomization analysis, the first place any reader should look is the choice of genetic variants. In this case, the genetic variants were chosen based on achieving a statistical significance threshold of *p*<0.001 in their association with BMI or waist circumference in the dataset under analysis. This selection rule raises a number of concerns (Figure).

First, winner’s curse bias, as genetic associations are typically overestimated in the dataset in which they were selected (5, 6). This affects genetic associations with the trait used for selection (typically the exposure), but also genetic associations with any variable correlated with the selection trait, such as the outcome. This typically biases Mendelian randomization estimates away from the null. Second, reverse causation, as these participants are the “oldest old”. It is possible that the genetic variants were not associated with increased BMI across the life course, but only in older age. This may be due to a downstream effect of a disease or frailty mechanism (7). Third, the genetic associations only achieve p-values of around 10^-4^ or 10^-5^. While the analysts considered fewer variants than assessed in a typical GWAS (around 20,000), a threshold of *p*<0.001 will lead to false positive findings. None of the 58 selected variants are significant for BMI at a multiply-corrected significance level of *p*<2.5 × 10^-6^, let alone achieving a genome-wide level of statistical significance. One of the 49 selected variants for waist circumference was significant at *p*<10^-5^ (rs2808521), although this variant has not been shown to be associated with any trait at a genome-wide significance level in any dataset in the GWAS Catalog (8). Given the amount of multiple testing in a genome-wide association analysis, this means that the genetic associations may be false positives. Fourth, this raises the possibility of weak instrument bias. If genetic variants are associated with the exposure at a genome-wide level of statistical significance, then weak instrument bias is typically ignorable (9). However, this is not the case for genetic variants associated at a weaker significance level. In a one-sample analysis (as in this analysis), weak instruments lead to biased estimates in the direction of the observational confounded association between the exposure and outcome (10). Fifth, the genetic variants are chosen based on statistical criteria only, and not according to their known function. While we sympathize with the difficulty of finding genetic predictors of traits in non-European populations, the choice of genetic variants in this analysis does not enable us to be confident that the instrumental variable assumptions are satisfied, and raises the strong possibility of bias.

An alternative approach for variant selection would be to initially start with genetic variants associated with BMI at a genome-wide level of significance in an external cohort (potentially a European ancestry dataset if an alternative Chinese dataset cannot be found), and filter out variants showing no association (say, p>0.01) in the dataset under analysis. While this approach also has drawbacks, it ensures that the genetic variants are robustly associated with BMI in adulthood in the external dataset, and mitigates against winner’s curse bias. A further alternative would be to use a cross-validation approach (11, 12), although given that no variants were robustly associated with the exposure in the full dataset, it is unlikely any robust associations would be found in a subset of the dataset.

Additionally, there is likely to be strong survival bias in the analysis. Genetic variants are assigned at conception, and so the “randomization” exploited in Mendelian randomization is valid for the population defined at conception. By only considering those alive at age 80, genetic associations will be subject to survival bias, an example of selection bias (also called collider bias) (13). Finally, non-linear Mendelian randomization analyses conducted using the residual stratification method are known to suffer from bias when the genetic effects on the exposure vary in the population (14). Alternative methods are recommended, although concerns have been raised about these methods (15). It is not clear whether the non-linear analyses presented were adjusted for age and sex, which is critical to mitigate bias.

Examining genetic associations in elderly individuals will always be difficult, as: 1) it is not clear whether such genetic associations hold across an individual’s whole life, 2) associations may be due to downstream effects of other traits (leading to reverse causation), and 3) associations will be subject to survival bias. Further, selecting genetic variants for Mendelian randomization based on associations in the dataset under analysis can lead to winner’s curse and weak instrument bias, particularly when the variants are chosen purely on statistical grounds, and the genetic associations do not reach conventional significance thresholds. The analyses conducted by the investigators showing that genetic predictors of BMI and waist circumference are associated with mortality in opposing directions are interesting, but these associations may reflect effects of selection or reverse causation rather than causal effects of BMI and waist circumference. The analyses presented by Lv *et al* are useful from the perspective of risk prediction, but cannot be interpreted as providing reliable evidence on causal relationships.

## Figures and Tables

**Figure:**
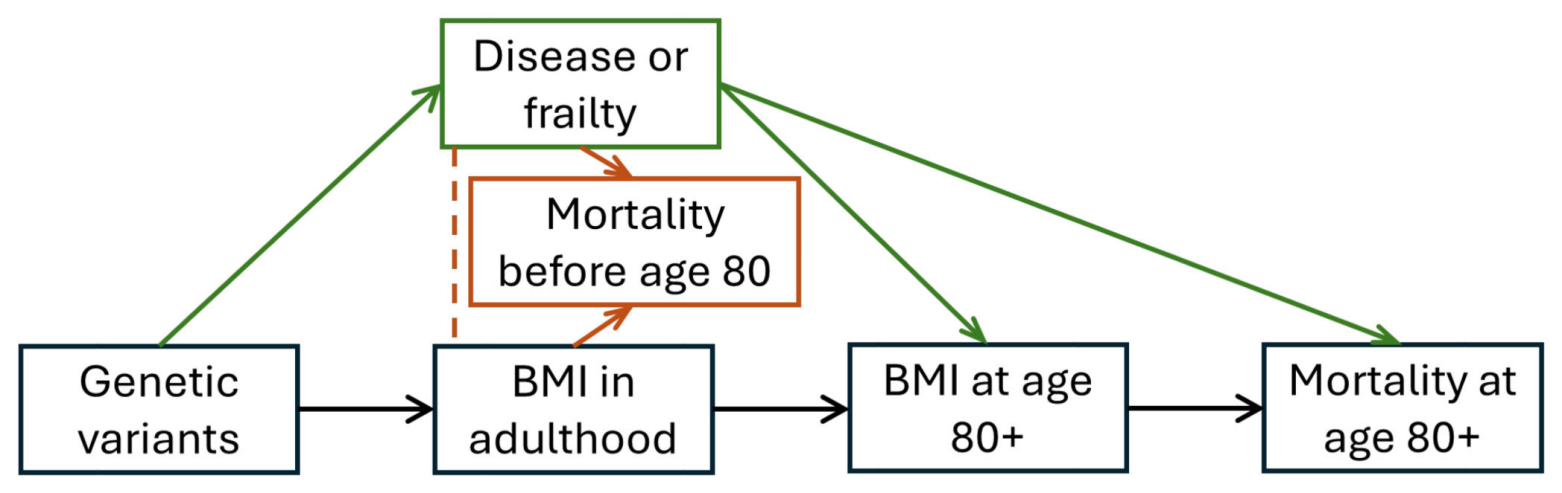
Schematic diagram indicating potential causal pathways by which the genetic variants may be associated with the exposure in late life. For a valid Mendelian randomization analysis, all causal pathways from the genetic variants to the outcome must pass via the exposure (black path). Genetic variants affecting BMI at age 80+ via a disease or frailty mechanism (green paths) may induce bias from reverse causation. Genetic variants influencing mortality before age 80 (orange path) may associate with BMI at age 80+ by survival bias, a particular example of selection bias (also called collider bias). As mortality before age 80 is a common effect of BMI on adulthood and disease, conditioning on survival until 80 will induce an association between BMI and disease (indicated by the orange dashed line). In both cases, there may be a pathway from the genetic variants to the outcome that does not pass via the exposure.
